# Research advances in the pathogenesis and therapeutic interventions of Helicobacter pylori and atrial fibrillation

**DOI:** 10.3389/fcvm.2025.1615707

**Published:** 2025-10-03

**Authors:** Zhaofen Wang, Haijuan Wang, Yi Yan, Peng Chang

**Affiliations:** ^1^ The Second Hospital & Clinical Medical School, Lanzhou University, Lanzhou, China; ^2^ Department of Cardiology, Lanzhou University Second Hospital, Lanzhou, 730030, China

**Keywords:** atrial fibrillation, helicobacter pylori, pathogenesis, inflammation, helicobacter pylori radication

## Abstract

Atrial fibrillation (AF) has emerged as a global health challenge in the 21st century. As the most common clinical arrhythmia, AF is characterized by high rates of recurrence, disability, and mortality, coupled with substantial healthcare expenditures. Despite significant advancements in AF management in recent years, the etiological drivers and pathogenic mechanisms remain incompletely understood in a subset of patients. Since the association between Helicobacter pylori (H. pylori) infection and non-gastrointestinal diseases was reported, H. pylori-associated non-gastrointestinal diseases have gradually attracted a lot of attention, especially in cardiovascular diseases. Nevertheless, studies on the relationship between H. pylori infection and AF are still limited, and the results are controversial. Rigorous large-scale studies are warranted to delineate the pathophysiological interplay between H. pylori colonization and AF pathogenesis, with particular emphasis on determining whether eradication therapy reduces AF-associated cardiovascular morbidity or enhances long-term patient outcomes. In view of this, the aim of this paper is to review the role of H. pylori in the occurrence and development of AF, to analyze the relationship between H. pylori-related cardiovascular diseases, non-cardiovascular diseases, and AF, and to explore the possible pathogenesis so as to provide new ideas and research directions for the treatment of AF, especially the intervention of idiopathic AF(iAF).

## 1 Introduction

AF, as a supraventricular tachyarrhythmia, is the ultimate manifestation of structural remodeling of the atrial area caused by several cardiovascular diseases. As the most common clinical arrhythmia, AF is independently associated with significantly reduced quality of life (QOL) and elevated mortality rates. This clinical burden stems from AF-induced systemic complications, including heart failure (HF), thromboembolic stroke, and cognitive decline, which collectively exacerbate disease progression and patient outcomes.

It is estimated that the incidence of AF has increased 50-fold in the past three years. In the United States, the incidence of AF is predicted to increase to 12.1 million by 2030 (1). In Europe, the number of AF patients aged ≥55 years was estimated at 9 million in 2010 and is predicted to reach 14 million by 2060 (2). Furthermore, epidemiological models forecast that Asia will have at least 72 million diagnosed AF cases by 2050, with an estimated 3 million individuals likely to experience AF-associated thromboembolic strokes (3).

Although the pathogenesis of AF is still being explored, the generally accepted pathogenesis of AF includes dysregulated calcium homeostasis, autonomic nervous system imbalance, and other factors that cause abnormal discharges in the myocyte sleeves within the pulmonary veins, atrial folding back circuits, early electrical remodeling, and structural remodeling due to advanced atrial fibrosis (4). In addition, a variety of factors, such as genetic factors, the renin-angiotensin-aldosterone system (RASS) activation, pre-existing cardiovascular comorbidities, aging, dietary patterns, and chronic inflammatory states. Therefore,the treatment of multiple underlying factors may help to improve the management of AF.

H. pylori was first isolated from the human stomach by Moran et al. in 1,899 (5). The bacterium derives its name from its predominant colonization of the gastric pyloric region and distinctive spiral morphology. Globally, the prevalence of H. pylori infection exceeds 50% (6). Its unique morphology and physiological capabilities enable it to penetrate the gastric mucosa and colonize the interstitial space barrier between mucus and sodium carbonate, allowing it to survive under the direct influence of gastric acid. By producing urease to break down urea, Helicobacter pylori generates CO_2_ and ammonia to neutralize gastric acid and create a mildly alkaline environment conducive to its survival (7). These synergistic mechanisms collectively enable H. pylori to evade gastric acid clearance and host immune surveillance, driving persistent chronic inflammation. Notably, only specific H. pylori strains express virulence determinants critical for pathogenicity, with cytotoxin-associated gene A (CagA) and vacuolar cytotoxin A (VacA) serving as hallmark virulence factors (8).

In recent years, H. pylori has been recognized as possibly being associated with AF, especially idiopathic AF (iAF, defined as AF without identifiable etiologies). Although H. pylori infection is not a primary risk factor for AF, it may influence the development of AF or exacerbate pre-existing AF through multiple mechanisms (9). Although the relationship between H. pylori infection and AF is still controversial, understanding these potential pathogenic mechanisms and their impact on AF may provide new ideas and strategies for the prevention and treatment of AF.

## 2 Method

We systematically searched relevant literature published up to February 2025 in the PubMed and Google Scholar databases. The search employed a combination of subject headings and free-text terms, with the core strategy as follows: (“Atrial Fibrillation” OR “AF” OR “Auricular Fibrillation”) AND (“Helicobacter pylori” OR “H. pylori” OR “pylori”). Additionally, the references of included studies were manually searched to identify potentially relevant articles. The inclusion criteria were: (1) observational studies (case-control, cross-sectional, or cohort studies), reviews, and meta-analyses that investigated the association between AF and H. pylori infection; and (2) studies providing complete data. The exclusion criteria included: (1) incomplete or unavailable data; and (2) conference abstracts and non-human studies. This review synthesizes current evidence on atrial fibrillation and Helicobacter pylori infection, with the aim of examining their correlation and exploring potential underlying mechanisms.

## 3 Association between H. pylori Infection and the onset and progression of AF

To date, the relationship between H. pylori and AF is not only controversial but the number of relevant studies is also relatively limited. And most of the existing studies are based on small samples (see Table 1).

**Table 1:** Characteristics of included studies on the association between H. pylori and AF.

Author	Association Between H. pylori and AF	H. pylori Diagnostic Method	Sample Size	Year
Montenero (10)	Strong association	Serum H. pylori IgG antibodies	104	2005
Badran (11)	CagA-positive H. pylori infection strongly correlates with AF in CAD patients	Serum H. pylori IgG antibodies	162	2007
Bunch (12)	Moderate association in high-risk CVD patients (non-significant after multivariable adjustment)	Serum H. pylori IgG antibodies	743	2008
Platonov (13)	No significant association in persistent AF	Serum H. pylori IgG antibodies	144	2008
Mujtaba (14)	No significant association	Biopsy	376	2008
Lunett (15)	No correlation	Serum H. pylori IgG antibodies	180	2009
Ki, (16)	Increased VacA seropositivity and decreased TGF-*β*1 in AF patients.	H. pylori or VacA antibody titers	96	2010
Franceschi (8)	Epidemiological link between CagA/VacA positive strains and AF in IA.	13C-UBT	104	2013
Wang (17)	H. pylori infection correlates with AF duration	13C-UBT	585	2015
Tetta (18)	No correlation	Meta-analysis	2,921	2019
Farah (9)	Significant association	Biopsy or UBT	180	2024

IA, idiopathic arrhythmia; UBT, urea breath test.

Frustaci et al. analyzed histologically the right atrial endocardial myocardial biopsy tissue from 12 patients with isolated atrial fibrillation (LAF) and compared it with 11 patients with Wolff-Parkinson-White syndrome (WPW). It was found that all LAF patients had abnormal atrial tissue, whereas the WPW controls were completely normal. Of these patients, 66% were consistent with a diagnosis of myocarditis, with lesions concentrated in the atria rather than the ventricles, and these structural changes within the atria suggested an inflammatory basis for AF (19). Further review of the literature shows that infection is one of the potential causative mechanisms of AF, the most common arrhythmia in the clinic. Infectious agents include several categories, such as bacteria, viruses, and fungi. Notably, among bacterial pathogens, H. pylori and Chlamydia pneumoniae exhibit the most robust associations with AF (20).

Montenero et al. explored the relationship between H. pylori and AF for the first time in a study of 45 patients with AF both paroxysmal and persistent AF and 45 healthy controls. The study found that patients with AF were commonly associated with gastric discomfort and that there was a strong association between H. pylori and AF. The mean H. pylori seropositivity was 97.2 IU/ml in patients with AF, whereas the mean H. pylori seropositivity in healthy controls was only 5.3 IU/ml. At the same time, the concentration of the reactive inflammatory marker, C-reactive protein (CRP), was significantly higher, with a mean CRP concentration of 8 mg/L in AF patients and a mean CRP concentration of 1 mg/L in healthy controls, suggesting that H. pylori infection may be responsible for the elevated CRP in AF patients. In addition, H. pylori seropositivity and CRP concentrations were significantly higher in patients with persistent AF compared to those with paroxysmal AF (100 IU/ml vs. 60.2 IU/ml, *p* = 0.027 and 9 mg/L vs. 7 mg/L, *p* = 0.041, respectively) (10). A study by Wang et al. demonstrated that a 13C/12C isotope ratio H. pylori *δ* value) ≥4‰ in H. pylori serves as an independent predictor of long-term AF (more than one year). The results of the study showed that the H. pylori*δ*value of 8.4‰in patients with long-term AF was significantly higher than that of 5.25‰ in patients with short-term AF (less than one year, and the difference was statistically significant (*P* < 0.001). Based on this, they concluded that the level of H. pylori infection was significantly elevated in patients with long-term persistent atrial fibrillation, and H. pylori infection can trigger an inflammatory response, which in turn is a significant risk factor for AF. Therefore, inflammation induced by H. pylori infection may be correlated with the development of AF (17). A study by Franceschi et al. of 54 patients with supraventricular or ventricular idiopathic arrhythmias (IA) and 50 healthy controls showed a significantly higher detection rate of CagA-positive strains, 65%, and a concomitant increase in the detection rate of VacA-positive strains, 74%, in patients with IA, as compared with healthy controls. Based on this result, they suggested that infection with cytotoxin-associated gene A (CagA) and vacuolar toxin A (VacA) -positive strains of H. pylori may be associated with the development of IA in patients with idiopathic arrhythmias who lack other cardiologic evidence (8). To further explore the relationship H. pylori and AF, Bunch et al. studied 943 patients with known AF status. They categorized the population into three groups according to age: <50 years, 51–70 years, and >70 years. In all three groups, results showed a higher prevalence of H. pylori infection in the AF group compared to the non-AF group (65% vs. 55%; *p* = 0.049). In particular, among those <50 years of age, the risk of AF was significantly higher in H. pylori-positive patients than in H. pylori-negative patients (8% vs. 0%). However, in the older age group of >70 years, despite the highest prevalence of AF, the risk of AF was only slightly increased in H. pylori-positive patients (17.5% vs. 15.4%). This result suggests that H. pylori infection may significantly increase the risk of developing AF in the younger population (12). In addition, if there is indeed a correlation between H. pylori and AF, it remains to be clarified whether H. pylori promotes the persistence of AF or persistent AF increases the susceptibility to H. pylori (17). A related study of 15 patients with paroxysmal AF found that the inflammatory markers high-sensitivity C-reactive protein (hs-CRP), interleukin-6 (IL-6), and tumor necrosis factor*α*(TNF-α) were still elevated even 24 h or even 2 weeks after resumption of rhythm. Therefore, they concluded that it was not the AF that caused the inflammation, but rather, it was the inflammation that caused the AF (21). It is hypothesized that inflammation plays an essential role in the development of AF.

In contrast, Tetta et al. explored the relationship between H. pylori and AF by means of a systematic evaluation and meta-analysis, in which they included a total of six retrospective studies covering 2,921 individuals. Of these, 956 patients (32.7%) had AF and 1965 (67.3%) were in normal sinus rhythm (NSR). Of the 956 patients with AF, 335 (35%) were positive for H. pylori. 643 (32.7%) of the 1965 patients with NSR were positive for H. pylori (*p* = 0.21). The cumulative risk ratio for developing H. pylori infection in patients with AF was 1.19 (95% confidence interval 1.08–1.41). Moreover, the difference in risk of AF found was relatively small [0.11 (SE = 0.04)]. Therefore, they concluded that there is no correlation between H. pylori and AF, and H. pylori cannot be used as a risk factor for AF (18). A cross-sectional study of 46 patients with AF by Marcus et al. also found no significant association between inflammatory markers (e.g., CRP, tumor necrosis factor-alpha, CD-40 ligand, monocyte chemotactic protein-1, and fibrinogen) and AF, except for IL-6 (22).

## 4 Possible pathogenic mechanisms linking H. pylori to AF

### 4.1 Inflammatory mechanisms

**Figure 1:**
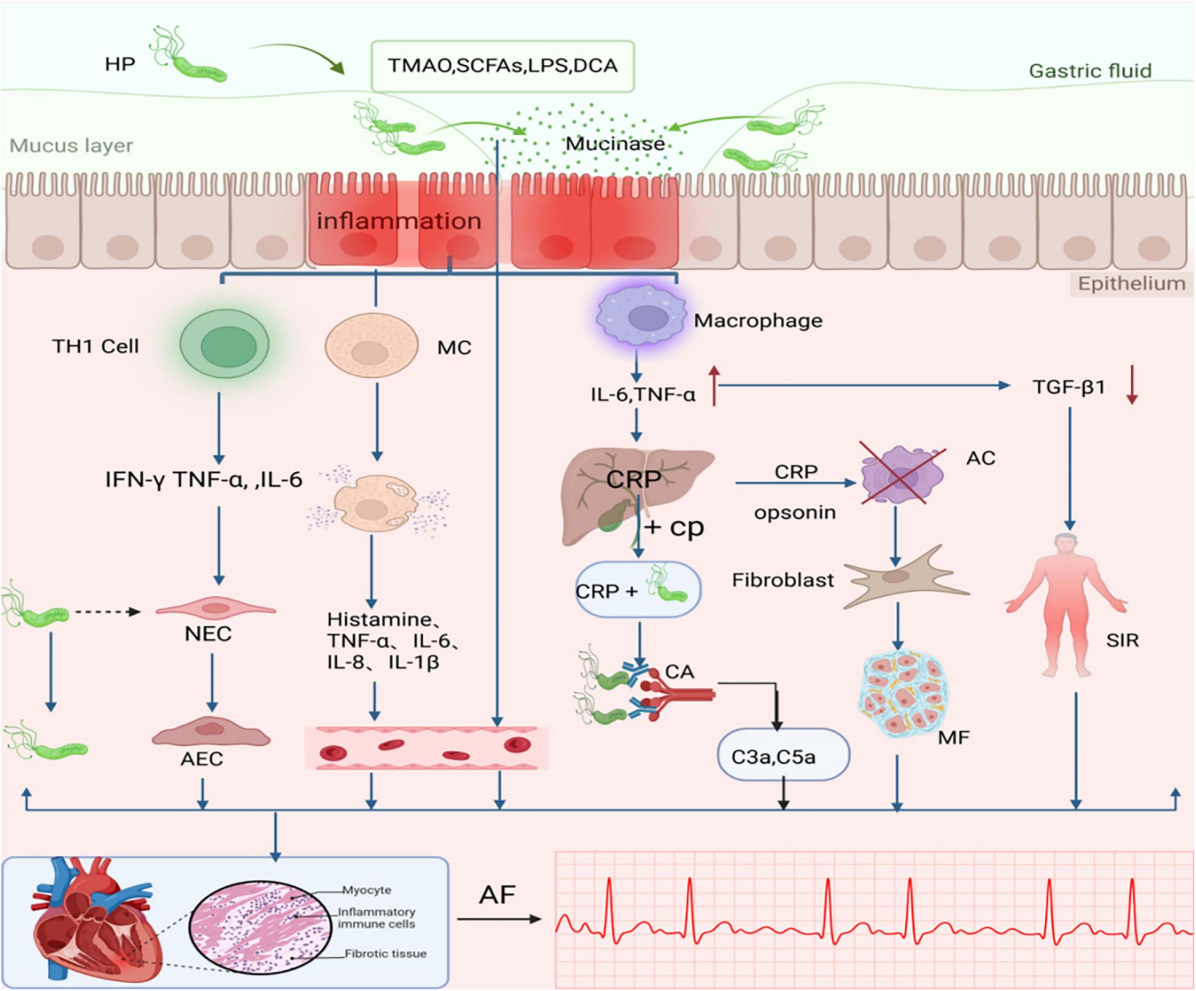
The association between H. pylori Infection-Induced Inflammatory Mechanisms and AF. One possible mechanism that causes AF after H. pylori infection is inflammation. H. pylori, Helicobacter pylori; MC, Mast cell; CP, Choline Phosphate; CA, Complement Activation; C3a C5a, Anaphylatoxin; AC, Apoptotic cell; NEC, Normal Endothelial Cell; AEC, Abnormal Endothelial Cell; SIR, Systemic Inflammatory Response; MF, Myocardial Fibrosis;CRP, vC-reactive protein; TMAO, trimethylamine N-oxide; SCFAs, short-chain fatty acids; LPS, lipopolysaccharide; BAs, bile acids. TNF, tumor necrosis factor; TGFβ1, Transforming growth factor β1; AF, atrial fibrillation; Dashed line with arrow: H. pylori is absent in the cell; Plus sign (+): indicates binding interaction;Cross mark (×): represents clearance mediated by apoptotic cells. An upward arrow signifies growth, while a downward arrow represents a reduction. Created in BioRender. Wang, Z. (2025), https://BioRender.com/s2f1zml, licensed under Academic License.

Inflammation induces myocardial fibrosis, leading to cardiac structural and electrical remodeling. Similarly, AF itself may trigger inflammatory responses through multiple pathways, which in turn perpetuate AF via these shared mechanisms. This bidirectional relationship supports the hypothesis that “AF begets AF” (23). A study by Yang, X. et al. on the relationship between systemic inflammatory markers and arrhythmias among 478,524 individuals from the UK Biobank cohort found that CRP levels were significantly positively associated with the risk of developing atrial fibrillation (AF). Compared to the reference group with CRP < 0.5 mg/L, the risk of AF for those with CRP > 10 mg/L was 1.33 (95% CI: 1.24–1.43). This association remained significant even after adjusting for all confounding variables (24) (See Figure 1).

Following H. pylori infection, local or systemic monocytes/macrophages are activated to produce cytokines such as TNF-α and IL-6, which further stimulate the synthesis of acute-phase proteins. IL-6, as the primary stimulatory factor, induces hepatic production of C-reactive protein (CRP). CRP recognizes foreign pathogens and phospholipid components of damaged cells by binding to phosphocholine. Upon ligand binding, CRP may activate the complement system or interact with phagocytes (25), thereby triggering localized myocardial inflammation and exacerbating cardiac injury. Dernellis et al. found that there was not even a correlation between CRP and AF in the absence of a high complement. They proposed that CRP is located in the atrial tissue and acts as an acute phase protein that promotes local complement activation (26). Complement-derived anaphylatoxins (e.g., C3a, C5a) enhance the release of inflammatory mediators triggered by antigen-antibody interactions, indicating that complement activation amplifies immune responses in cardiac inflammation (27). Thus, increased complement components may be one of the potential factors promoting the development of AF in patients with high CRP.

The primary trigger of AF is the abnormal electrical activity originating from the pulmonary vein orifices (28). The high rate of electrical activity in AF may overload the myocytes with calcium ions, and in some cases, the calcium overload leads to apoptosis of the atrial myocytes (29). As an acute-phase protein, CRP facilitates the clearance of apoptotic cells (30), and these apoptotic regions are subsequently substituted by fibrotic tissue. This low-grade inflammation and fibrosis work together to disrupt the cardiac electrical conduction system and underlie the persistence of AF (31).

In addition, not only are there elevated inflammatory markers such as CRP and proinflammatory cytokines (e.g., TNF-α, IL-6) in H. pylori-infected patients, but reduced transforming growth factor-beta 1 (TGF-β1) concentrations may mediate systemic inflammatory responses, thereby increasing the risk of AF (16). TGF-β1 exerts cardioprotective effects by suppressing pathogenic Th1-mediated immune responses (32). Therefore, low TGF-β1 concentrations, particularly in particularly in the context of CagA-positive H. pylori infection, may increase the risk of AF recurrence (16, 33).

Recent studies have demonstrated that H. pylori can activate mast cells (MCs) and promote the release of inflammatory mediators (34). Concurrently, H. pylori infection compromises intestinal barrier integrity, allowing inflammatory mediators and gut-derived molecules (e.g., microbial metabolites) to enter systemic circulation, thus causing local or systemic inflammation (35). This cascade of events provides a pathological basis for the initiation and progression of atrial fibrillation(AF).

Furthermore, H. pylori infection stimulates the development of a Th1 cell-mediated inflammatory response (6). Although this immune response is intended to eliminate intracellular pathogens, H. pylori does not reside within host cells, rendering the Th1 response ineffective against the bacteria while exacerbating epithelial damage. Thus, the presence of H. pylori leads to long-term chronic inflammation and cellular damage (36). Rossi et al. suggested that latent H. pylori infection or the persistence of bacterial antigens induces prolonged cardiac inflammation, which can lead to cardiac tissue remodeling and fibrosis (37).

In addition to the aforementioned pro-inflammatory mechanisms, H. pylori infection also elicits anti-inflammatory responses, which contribute to its ability to persist chronically in the human host without triggering excessive immune reactions, instead establishing long-term chronic inflammation (38). Specifically, following H. pylori infection, the bacterium can activate various T-cell subsets, leading to the systemic release of cytokines. Among these cytokines, pro-inflammatory factors (e.g., IL-17, IL-21, IL-22, and IL-23) interact with anti-inflammatory factors (e.g., IFN-γ, IL-1β, IL-7, IL-10, and IL-18) (39). This pro-inflammatory and anti-inflammatory regulatory mechanism downregulates the immune response, thereby modulating the adaptive response to prevent excessive immune activation. Compared to CagA-negative strains, CagA-positive strains exhibit these effects more prominently (40).

### 4.2 Cytotoxin-associated gene A (CagA)

**Figure 2:**
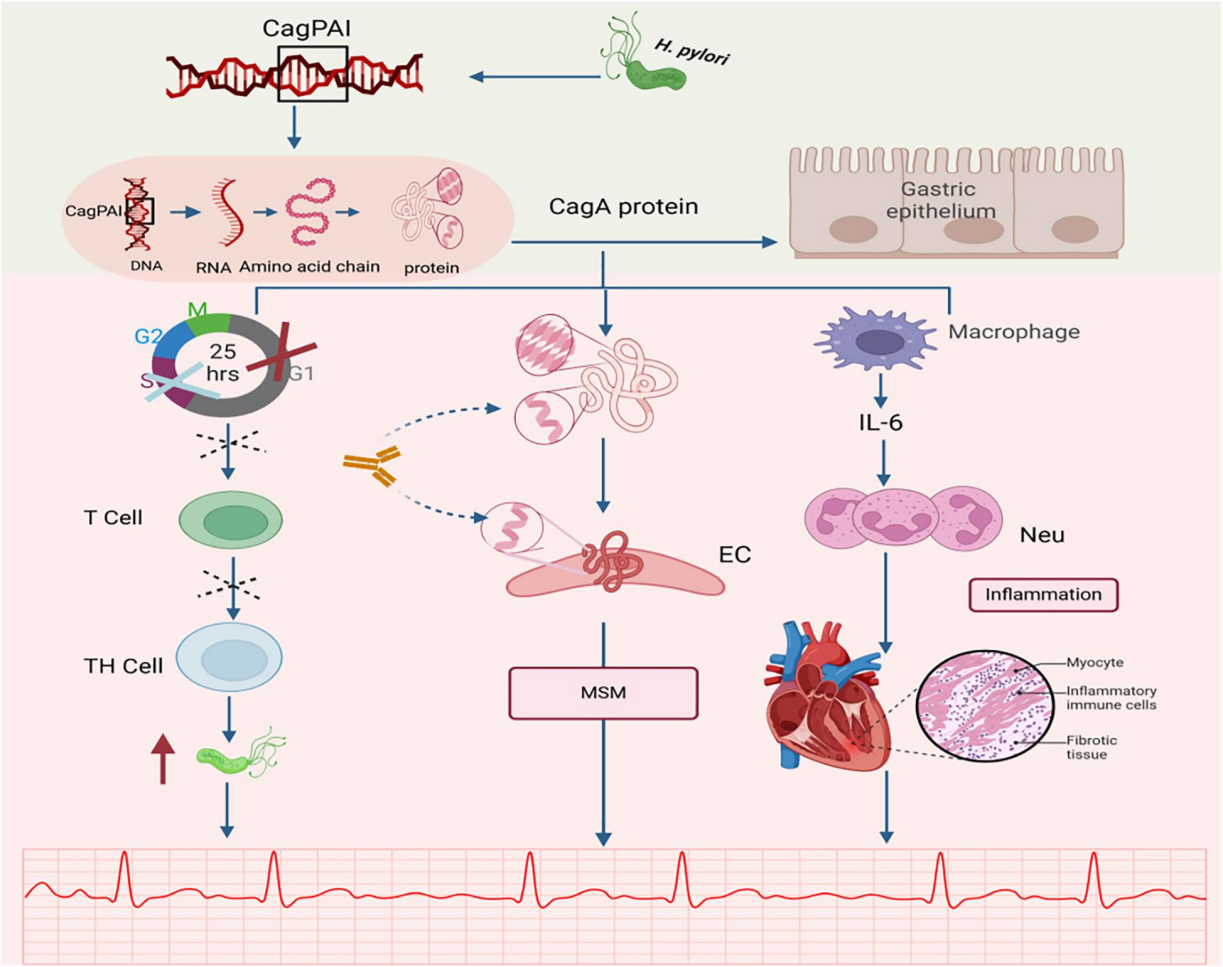
Relationship between CagA and AF. CagA, a major virulence factor encoded by H. pylori, has been identified as a potential pathogenic mechanism for inducing atrial fibrillation (AF). CagA, cytotoxin-associated gene A; CagAPAI, cag pathogenicity island; MSM, mechanism of molecular simulation; EC, endothelial cell; Neu, neutrophil; Cross mark (×): Indicates G1/S phase cell cycle arrest; Dashed cross: Represents inhibition of T cell proliferation. Created in BioRender. Wang, Z. (2025), https://BioRender.com/qc43cc1, licensed under Academic License.

Badran et al. determined the seroprevalence of CagA by enzyme-linked immunosorbent assay (ELISA) in 185 patients with coronary artery disease(CAD), with or without AF, and 80 healthy subjects. The results revealed that CagA seropositivity was significantly higher in the CAD group (49.7%, 92/185) than in the control group (11.3%, 9/80) (*P* < 0.004). Moreover, regarding CagA-positive strain prevalence, it was significantly prevalent in the atrial fibrillation group compared with the sinus group [52 (63.4%) vs. 40 (38.8%)] (OR 3.59, 95% CI 1.87–6.94, *P* < 0.001) and control group [21 (26.3%), OR 5.49, 95% CI 2.3–12.96, *P* < 0.001]. Thus, they concluded that CagA was strongly correlated with coronary artery disease and that CagA was significantly elevated in coronary artery disease compared to controls, with the strongest correlation in patients with AF (11) (See Figure 2).

CagA is a high-molecular-weight antigen of H. pylori. H. pylori strains are classified into CagA-positive and CagA-negative subgroups based on the presence or absence of the CagA gene, which encodes the CagA protein (41). The CagA gene is a specific gene sequence that is present in a genomic DNA fragment of approximately 40 kb in length. This particular DNA fragment is named cag pathogenicity island (cag PAI). Notably, cag PAI can transport CagA to the cytoplasm of gastric epithelial cells, a process central to H. pylori-mediated pathogenesis (41, 42). Wang et al. proposed that CagA is released during H. pylori infection, which stimulates the secretion of interleukins from gastric epithelial cells and induces neutrophil chemotaxis and infiltration, thereby triggering an inflammatory response (43).

Additionally, molecular mimicry exists between the CagA protein (encoded by the CagA gene) and cardiovascular peptides in normal vascular smooth muscle cells and endothelial cells (44), which may lead to antigenic cross-reactivity (44), resulting in the so-called molecular mimicry mechanism. This suggests that gastric injury caused by H. pylori infection may trigger a molecular mimicry mechanism, providing a novel potential explanation for the pathogenesis of cardiovascular diseases, including AF (45).

Emerging evidence suggests that H. pylori functions not only as an inflammatory mediator but also as an immunomodulator. Its virulence factor, CagA, partially mimics the immunosuppressive drug FK506 by inducing local immune suppression. In addition, CagA induces host cell cycle arrest at the G1/S phase. This process not only directly affects epithelial cells but, more critically, perturbs T cell activation and proliferation. As activated T cells play a central role in coordinating both pro-inflammatory Th1 and anti-inflammatory Th2 responses, their functional suppression disrupts the overall balance of the adaptive immune response. This shift promotes a Th2- and regulatory T cell (Treg)-skewed response, marked by elevated secretion of molecules such as IL-10, while concurrently weakening Th1 activity. The resulting Th1/Th2 imbalance leads to a generalized immunosuppressive state, which may facilitate persistent *H. pylori* colonization and survival—potentially elevating the risk of atrial fibrillation (12, 16, 46).

### 4.3 Interplay between ion pumps

**Figure 3:**
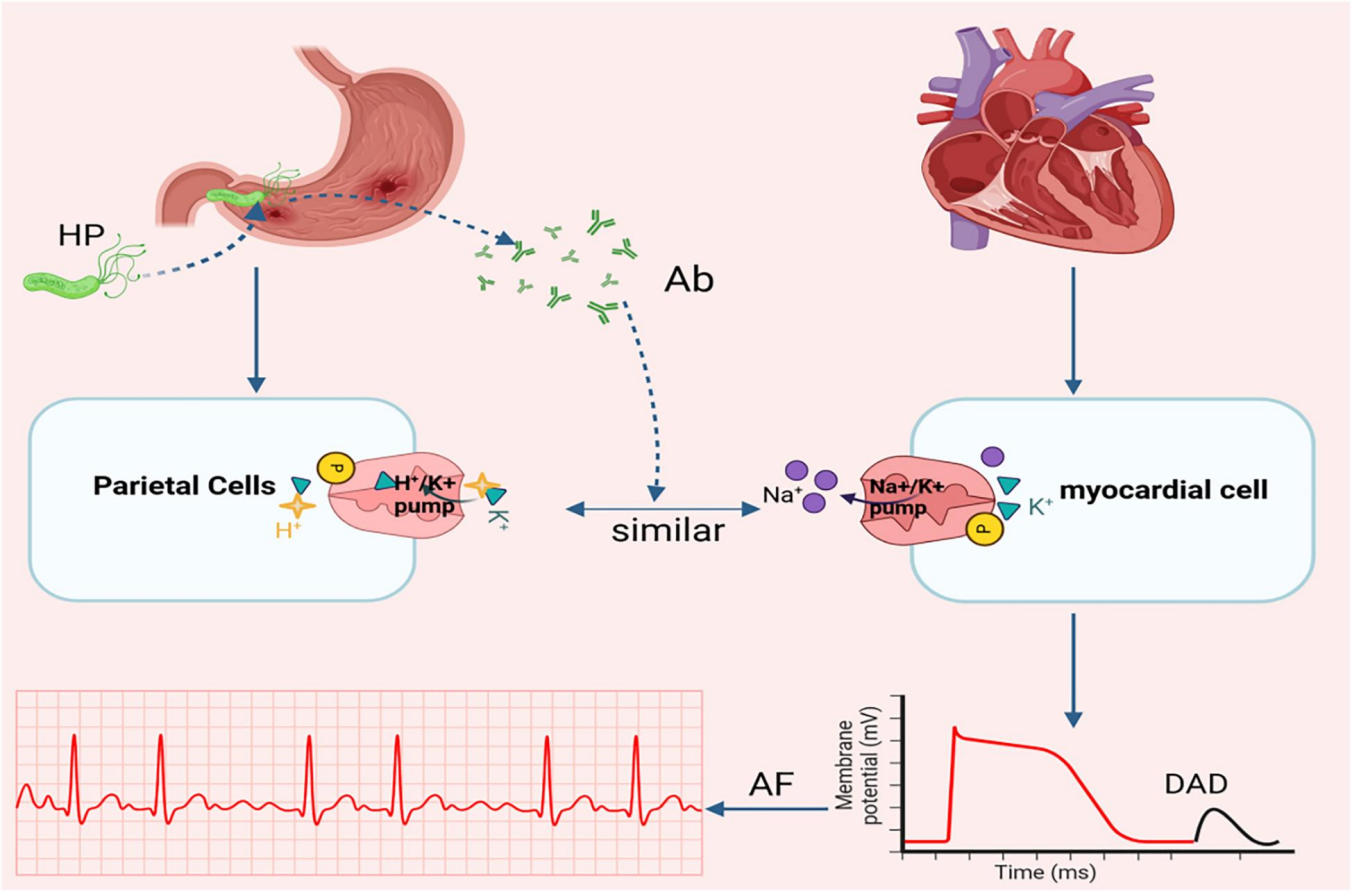
Relationship between ion pump and AF. Another potential mechanism linking H. pylori infection to AF involves molecular mimicry between the gastric H+/K+ ATPase (abundantly expressed in parietal cells) and the Na+/K+ ATPase on myocardial cell. pylori, Helicobacter pylori; Ab, Antibody; DAD, delayed afterdepolarizations; AF, atrial fibrillation. Created in BioRender. Wang, Z. (2025), https://BioRender.com/vpdi96d, licensed under Academic License.

In addition to the inflammatory response, another critical evidence linking Helicobacter pylori H. pylori infection to AF originates from a study by Andrew et al. They demonstrated structural homology between the gastric H^+^/K^+^-ATPase and the cardiac Na^+^/K^+^-ATPase (47), characterized by two key similarities: (1) Both ion pumps rely on a 35-kDa glycoprotein subunit for catalytic activity. (2) Both require ATP hydrolysis to maintain ionic homeostasis. However, H. pylori infection may induce autoantibodies targeting the gastric H^+^/K^+^-ATPase. These antibodies may unintentionally act on the heart and affect the normal function of the Na^+^/K^+^-ATPase in the heart, leading to myocardial damage, delayed afterdepolarization, and premature atrial contractions of which elevate AF risk (10) (see Figure 3).

### 4.4 Dysregulation of gut microbiota

In recent years, the concept of the “gut-heart axis” has emerged as a new direction in cardiovascular research (48). This bidirectional communication involves interactions between the gastrointestinal tract and the cardiovascular system through neural, hormonal, metabolic, and immune pathways.Together, these pathways influence ion channel expression, myocardial excitability, conduction properties, and autonomic nerve tone-key factors in arrhythmogenesis (49).

On the other hand, a central regulator within this axis is the gut microbiota, the vast community of microorganisms residing in the human body that contributes to a range of host physiological processes, including nutrient absorption, immune development, and metabolic homeostasis (50). Disruption of the gut microbial ecosystem, often termed dysbiosis, has been increasingly linked to various cardiovascular diseases, such as hypertension, coronary artery disease, heart failure, and particularly arrhythmias (49).

Helicobacter pylori may disrupt the gut microbiota, leading to an imbalance in the gut-heart axis. Such dysbiosis has been associated with the onset and progression of atrial fibrillation, suggesting a novel pathway through which H. pylori could indirectly affect cardiac function via its influence on the gut microbiome (18).

### 4.5 Other mechanisms

According to the guidelines for the treatment of patients with atrial fibrillation, anticoagulation is explicitly recommended for stroke prevention. One study showed that 56% of anticoagulated AF patients had H. pylori infections, including those without dyspepsia symptoms (51). In addition, a retrospective cross-sectional study involving 125 patients with dyspepsia found that although the incidence of gastritis and H. pylori infection was similar in patients treated with direct oral anticoagulants (DOACs) as in those not using DOACs, the incidence of H. pylori infection, upper gastrointestinal ulcers, and gastritis was significantly higher when DOACs were used for more than 12 months (52). Based on these findings, we hypothesize that prolonged anticoagulation may elevate H. pylori infection rates in AF patients, thereby contributing to and maintaining the development of AF. However, this speculation still needs to be validated by further studies.

Studies have demonstrated that H. pylori antibodies can be directly detected in arterial blood (53). The prolonged *in vivo* persistence of H. pylori may be closely associated with monocyte-mediated immune responses, which trigger elevated levels of inflammatory factors (e.g., interleukin, tumor necrosis factor) in the bloodstream, ultimately promoting vascular fibroblast proliferation (54). Another hypothesis of interest is that H. pylori may be specifically selective for the pulmonary veins and other veins (e.g., vena cava, coronary sinus) through the blood circulation. However, H. pylori was not detected in the blood, so how H. pylori reaches the target vessels remains difficult to explain. However, since H. pylori has not been detected in blood samples, the mechanism by which it reaches its vascular targets remains unresolved.

In addition to the mechanisms above, it is hypothesized that circulating H. pylori may interact with monocytes in the bloodstream, potentially stimulating cardiac fibroblast proliferation. This process could initiate cardiac fibrosis and promote structural remodeling of the heart, ultimately contributing to the pathogenesis of atrial fibrillation. However, this hypothesis warrants further experimental validation through systematic *in vitro* and *in vivo* investigations.

## 5 The association between H. pylori-related cardiovascular disease and AF

In recent years, it has been found that H. pylori infection may be associated with a variety of extra gastric diseases, including cardiovascular diseases, and in particular, there have been more advances in the research between atherosclerotic cardiovascular diseases and H. pylori infection and the available evidence suggests that diseases such as coronary artery disease, stroke, and hypertension are often coexisting with or are risk factors for AF (55).

A study by Shmuely et al. found that H. pylori infection induces metabolic dysregulation of key lipid components, including low-density lipoprotein (LDL), high-density lipoprotein (HDL), and total cholesterol *in vivo* (56). The prevalence of hypertriglyceridemia, high cholesterol, elevated LDL levels, and reduced HDL levels was significantly higher in H. pylori-infected patients than in uninfected individuals (6). In addition, H. pylori infection decreases the absorption of vitamin B12 and folate (6), affects the role of the one-carbon units in DNA methylation, interferes with amino acid and lipid homeostasis, ultimately affecting DNA synthesis and cellular inflammation, and increases the synthesis of homocysteine and fat (57). All these changes lead to damage to the vascular endothelial injury, thereby initiating endothelial dysfunction and accelerating the pathogenesis of atherosclerosis. In addition to these effects, chronic H. pylori infection may exacerbate atherogenesis through multiple mechanisms, including disturbances in glucose metabolism, ammonia-induced sodium retention, direct vascular invasion, and molecular mimicry (58). For example, a study evaluating the relationship between H. pylori infection and coronary artery calcification (CAC) scores revealed a significant positive correlation, and this relationship was independent of traditional atherosclerosis risk factors (59). A study by Badran et al. of 185 patients with coronary artery disease (which included patients with or without AF) and 85 healthy controls. Their findings demonstrated that CagA seropositivity was significantly elevated in CAD patients with concomitant AF. Furthermore, the CagA-positive subgroup exhibited higher C-reactive protein (CRP) levels, leukocytosis, and more pronounced left atrial enlargement, suggesting that CagA-positive CAD patients may face an increased risk of AF development (11, 60). Abolbashari et al. noted that atherosclerosis is a known risk factor for AF and that atherosclerosis itself is an independent risk factor for AF (61).

Regarding cerebrovascular outcomes, Doheim et al. investigated the association between H. pylori infection and stroke by systematic evaluation and meta-analysis and found that H. pylori infection significantly increased the risk of stroke, especially in CagA-positive and ¹³C-urea breath test results breath test-positive patients (62). While stroke and AF are mutual risk factors, van Bree et al. reported that although stroke is not a direct cause of AF, the risk of new-onset AF is significantly increased after stroke (63).

Emerging evidence further substantiates the association between H. pylori infection and the onset and progression of hypertension has been much reported. Several studies have demonstrated that systolic blood pressure and mean arterial pressure were significantly higher in H. pylori individuals compared to uninfected controls (64). A high-sodium diet is one of the critical pathogenic mechanisms of hypertension, which not only promotes H. pylori aggregation and colony formation but also increases the binding of mucocyte mucin to H. pylori on the inner surface of the stomach, decreases the anti-H. pylori glandular mucocyte mucin and disrupts the bicarbonate gel layer of the gastric mucosa (65), which further increases H. pylori colonization and triggers cardiovascular disease. Wan et al. conducted a large-scale epidemiological study involving 5,246 adult participants from health management centers to explore the association between H. pylori infection and the prevalence of hypertension in Chinese adults, and they found that H. pylori infection was positively associated with the prevalence of hypertension (64). Lawler et al. concluded that hypertension is one of the most important risk factors for the development of AF worldwide and that approximately 25% of patients with AF are associated with hypertension (66).

Myocardial infarction (MI) is a fatal cardiovascular disease for which H. pylori infection is considered a potential risk factor (67). A follow-up study showed that H. pylori infection was present in one-fifth of patients at 3-month follow-up after acute myocardial infarction (68). Notably, a bidirectional relationship exists between MI and AF, and AF may directly trigger myocardial infarction through mechanisms such as coronary thromboembolism and insufficient coronary perfusion (69). Conversely, MI also promotes AF development, especially after acute MI, which is associated with a higher risk of AF and an 87% higher mortality rate compared with patients with persistent AF (70).

In summary, H. pylori infection is closely associated with various cardiovascular diseases (e.g., atherosclerosis, hypertension, myocardial infarction, stroke) and influences the development of AF through multiple mechanisms. In-depth research into the relationship between H. pylori and AF not only enhances our understanding of AF pathogenesis but may also offer novel strategies for its future prevention and treatment.

## 6 The association between H. pylori-related non-cardiovascular diseases and AF

In addition to cardiovascular disease, H. pylori infection has been associated with a variety of non-cardiovascular conditions that are also strongly linked to the development of AF. Studies have shown that H. pylori infection is independently associated with insulin resistance (IR), metabolic dysfunction-associated fatty liver disease (MAFLD) (71), and obesity, which contribute to the onset and progression of AF and its complications (e.g., stroke) through multiple mechanisms (34).

H. pylori infection activates the nuclear factor kappa B (NF-*κ*B)-associated inflammatory signaling cascade, which disrupts insulin metabolism, leading to insulin resistance (72, 73). In turn, insulin resistance is associated with AF and doubles the odds of cardiovascular disease, including stroke (74). In addition, glucose intolerance and insulin resistance not only lead to diabetes but also cause structural abnormalities of the left atrium (LA) and left ventricle (LV), establishing a pathological substrate for AF (75).

It has been proposed that H. pylori infection induces elevated levels of plasminogen activator inhibitor-1 (PAI-1) and other prothrombotic and inflammatory factors, thereby promoting the development of nonalcoholic fatty liver disease (NAFLD)-related cardiovascular diseases (76). A study by Cai et al. found that NAFLD significantly increased the risk of AF, and although the correlation was attenuated after adjusting for metabolic risk factors, the risk was still high compared to patients not patients with NAFLD and still had a higher risk (77). In addition, H. pylori-associated galectin-3 has been implicated in the pathophysiological mechanisms of NAFLD (33), and galectin-3, a biomarker of fibrosis, has been associated with atrial remodeling in patients with AF and plays a critical role in AF pathogenesis (33, 78).

A clear association exists between H. pylori infection and obesity. Studies have demonstrated that H. pylori infection is positively correlated with body mass index (BMI). Obese individuals may indirectly contribute to the initiation and perpetuation of AF hypertension, diabetes, metabolic syndrome, coronary heart disease, obstructive sleep apnea, and neurohormonal activation (79). Additionally, obesity can also cause left atrial enlargement, myocardial fibrosis, and conduction abnormalities.These structural and electrophysiological alterations collectively contribute to AF development and persistence (80).

H. pylori infection may also impair renal function. A meta-analysis showed that approximately 48% of patients with chronic kidney disease (CKD) are infected with H. pylori (81). A study demonstrated a positive correlation between urinary albumin-to-creatinine ratio (UACR) and H. pylori infection, with these parameters improving following H. pylori eradication. This suggests that H. pylori infection may be a potential risk factor for chronic renal injury in patients with peptic ulcer. Additionally, decreased glomerular filtration rate (GFR) and increased UACR were significantly associated with the development of AF (82). Watanabe et al. highlighted a bidirectional relationship between CKD and AF, sharing common risk factors and pathogenic mechanisms (83).

In summary, H. pylori is not only associated with cardiovascular diseases, but also closely linked to various non-cardiovascular conditions(e.g., IR, MAFLD, obesity, CKD, etc.). These comorbidities are independently associated with an elevated risk of AF. Therefore, further study of the relationship between H. pylori and these diseases is critical for advancing AF prevention and therapeutic strategies.

## 7 Impact of H. pylori eradication on AF

The necessity of anti-H. pylori therapy and optimal treatment strategies for AF patients with H. pylori co-infection remain subjects of ongoing debate.A study reported on a 51-year-old male patient who was hospitalized for AF combined with gastritis and positive for H. pylori, who had been treated with electrical cardioversion and pulmonary vein isolation on several occasions with poor results. Notably, without additional AF-specific interventions, sinus rhythm was restored within months following H. pylori eradication (47). This case suggests that H. pylori eradication may be necessary for AF patients with concurrent H. pylori infection. However, it may also be a result of the chance, that large-scale clinical studies with long-term follow-up are required to validate whether H. pylori eradication confers definitive clinical benefits in AF management.

Currently, there are no established guidelines or clinical studies addressing whether atrial fibrillation (AF) patients should undergo anti-Helicobacter pylori (HP) therapy. Baty et al. suggest that clinicians should prioritize early screening for HP infection in elderly individuals to mitigate potential clinical risks and help eliminate barriers to the use of direct oral anticoagulants (DOACs) in older AF patients (84). Lin et al., on the other hand, propose that proton pump inhibitors (PPIs) may act as inhibitors of AF. They argue that PPIs prevent AF recurrence by preserving glutathione, which functions as an antioxidant, and by suppressing inflammation (85). In a small-scale controlled study, Stöllberger et al. investigated 18 patients (6 women, 12 men, aged 39–69) with paroxysmal vagal atrial fibrillation—occurring primarily during sleep and rest—who also had esophagitis. Their findings indicated that PPI treatment for esophagitis not only alleviated esophageal inflammation but also completely eliminated or reduced the frequency of AF episodes (86). Gerson et al. studied three patients with gastroesophageal reflux disease who underwent simultaneous Holter monitoring and 24 h pH testing. All patients exhibited a reduction in arrhythmia symptoms following acid-suppressive therapy (87).

On the other hand, emerging evidence suggests that eradication of H. pylori may adversely affect AF, especially in asymptomatic H. pylori-positive individuals, and the potential side effects of eradication therapy remain unclear and may lead to increased risk of antibiotic resistance and abuse. A study conducted in Taiwan explored the relationship between gastroesophageal reflux disease (GERD) and AF. It found that the use of PPIs was associated with an elevated risk of AF (87). Kim et al. evaluated the long-term clinical consequences of failed H. pylori eradication in patients with AF. Their findings indicate that H. pylori infection may induce vascular endothelial cell damage, contributing to vascular dysfunction and that unsuccessful eradication is associated with elevated local or systemic oxidative stress—a well-established risk factor for AF. They found that AF patients with H. pylori treatment failure had a higher incidence of cardiovascular events, including atrial and ventricular arrhythmias. In addition, treatment failure for H. pylori infection is an independent risk factor for stroke, thus increasing the likelihood of neurological complications (e.g., painless motor weakness, hemiparesis, and cognitive impairment) in this population (88).

In summary, current studies on Helicobacter pylori (H. pylori) eradication therapy in AF patients with concurrent H. pylori infection remain limited, particularly regarding three critical aspects: (i) the necessity of eradication therapy, (ii) optimal drug regimens, and (iii) potential adverse effects associated with unsuccessful eradication attempts. Therefore, long-term, prospective, randomized controlled trials (RCTs) are warranted to elucidate the specific impact of H. pylori eradication on AF pathogenesis and progression, thereby generating robust evidence to guide clinical decision-making.

## 8 Current research limitations in AF patients with H. pylori co-infection

Current studies on the association between H. pylori and AF still have significant limitations: first, Most studies are single-center with small sample sizes, limiting the generalizability and reliability of conclusions.; second, variations in detection methods (e.g., the inability of serological antibody tests to distinguish active infection from prior exposure, differing specificity among breath tests, urease tests, and biopsies) may introduce bias.; Third, Whether H. pylori directly contributes to AF pathogenesis or indirectly interacts via mechanisms like inflammatory cytokines and oxidative stress remains unverified; fourth, Insufficient adjustment for age, coexisting cardiovascular diseases, H. pylori strain variations, and traditional AF risk factors. Furthermore, the lack of long-term follow-up data and dynamic inflammatory marker monitoring also made it challenging to elucidate the role of H. pylori infection in the development of AF. Of course, the most important point is that there is currently a lack of large-scale, prospective, randomized controlled clinical trials to directly validate the causal relationship between Helicobacter pylori infection and atrial fibrillation. Existing research is predominantly based on observational designs, in which potential confounding factors (such as age, hypertension, or socioeconomic status) may partially explain the observed associations. Therefore, future studies should focus on conducting large-sample, multicenter cohort studies, establishing a standardized H. pylori detection system, and further exploring relevant molecular mechanisms to overcome the current research limitations and provide more reliable evidence for clinical intervention.

## 9 Conclusion

This review systematically described the potential association between H. pylori and AF and its possible pathogenesis and explored the clinical implications of targeting H. pylori for AF management. As a prevalent gastrointestinal pathogen, H. pylori is not only implicated in digestive disorders but may also contribute to cardiovascular pathologies through multiple pathways, including systemic inflammation, CagA-mediated virulence, and interactions with gastric potassium-hydrogen ATPase pumps. While the H. pylori-AF correlation remains debated, emerging evidence positions H. pylori as a modifiable risk factor for AF. In clinical practice, H. pylori screening is recommended for people at high risk of AF (e.g., regions with elevated H. pylori prevalence and idiopathic AF cases) for early identification of potential risks. In addition, the effect of H. pylori eradication on AF still needs to be explored in depth: future studies need to clarify the causal relationship between H. pylori infection and AF, assess whether H. pylori eradication therapy can reduce the risk of new-onset AF or improve the prognosis of patients with AF, and validate its time-sensitive effect through long-term follow-up.

Suppose further studies confirm that H. pylori infection is an independent causative factor of AF. In that case, intervention strategies targeting H. pylori (e.g., antibiotic eradication therapy) are expected to provide new ideas for AF management. This finding may introduce a novel adjunctive therapeutic approach to conventional treatments for atrial fibrillation, such as antiarrhythmic drugs or catheter ablation. When combined with guideline-recommended treatment strategies, Helicobacter pylori eradication may contribute to a reduction in AF recurrence, alleviate the overall disease burden, and thereby further enhance the quality of life for patients.
